# Lipid Metabolism in Central Nervous System Diseases: Pathological Mechanisms and Therapeutic Advances

**DOI:** 10.1002/cns.71109

**Published:** 2026-08-25

**Authors:** Jiayan Yuan, Shixue Huang, Kun Jiao, Shu Yang, Xuhui Ge, Wei Liu, Wei Wang, Haoming Shu, Xuhui Zhou

**Affiliations:** ^1^ School of Health Science and Engineering University of Shanghai for Science and Technology Shanghai China; ^2^ Department of Orthopedics, Changzheng Hospital The Second Affiliated Hospital of Naval Medical University Shanghai China; ^3^ Department of Stress Medicine, Faculty of Psychology Naval Medical University Shanghai China; ^4^ Department of Orthopedics, Shanghai General Hospital Shanghai Jiao Tong University School of Medicine Shanghai China

**Keywords:** lipid metabolism, neurodegeneration, therapeutic advances, traumatic CNS injuries

## Abstract

**Background:**

Lipids are fundamental components of the central nervous system (CNS) structure and are essential for maintaining physiological homeostasis. Dysregulation of lipid homeostasis is a critical mechanism driving the pathogenesis and progression of classic CNS diseases, including neurodegenerative disorders, traumatic injuries, and cerebrovascular diseases. These metabolic disturbances trigger pathological processes such as abnormal protein aggregation, neuroinflammation, and lipid peroxidation, creating a vicious cycle that exacerbates disease progression. While targeting lipid metabolism offers a novel therapeutic strategy, its clinical translation faces significant hurdles.

**Scope of Review:**

This review systematically summarizes the pathological roles and molecular mechanisms of lipid metabolism dysregulation across six representative CNS disorders: Alzheimer's disease (AD), Parkinson's disease (PD), spinal cord injury (SCI), traumatic brain injury (TBI), stroke, and intracerebral hemorrhage (ICH). These conditions were selected to cover a spectrum of pathologies, including protein‐aggregation neurodegeneration, posttraumatic secondary injury, and cerebrovascular damage. The article outlines recent advances in therapeutic interventions targeting lipid metabolism and dissects both the shared and disease‐specific regulatory mechanisms within the CNS.

**Key Challenges:**

Despite the therapeutic potential, several challenges impede the development of effective lipid‐targeting treatments. These include the incompletely defined mechanisms underlying dynamic lipid changes across different disease stages, insufficient efficiency of drug delivery across the blood–brain barrier, and the limited efficacy of single‐target interventions. This review addresses these existing hurdles and proposes potential solutions to overcome them.

**Conclusion:**

Targeting lipid metabolism represents a promising frontier for treating CNS diseases. By providing a comprehensive reference on the metabolic underpinnings of these disorders and addressing current research bottlenecks, this review aims to lay the groundwork for the development of precise, targeted therapeutic strategies in future clinical applications.

## Graphical abstract

Dysregulated lipid metabolism in CNS diseases: core mechanisms and targeted interventions. Lipid metabolism dysregulation participates in the pathological progression of neurodegenerative diseases, traumatic CNS injuries and cerebrovascular diseases by mediating key pathological processes including abnormal protein aggregation, neuroinflammation, lipid peroxidation, and demyelination. Targeting lipid metabolism may provide a novel therapeutic direction for CNS diseases. This figure was created by www.Biorender.com.

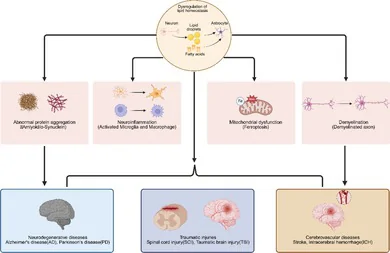

## 1 Introduction

The central nervous system (CNS) relies on precise networks of metabolism and signal regulation for its structural and functional integrity, among which lipid metabolism represents an indispensable key component [1]. The brain is the most lipid‐rich organ in the human body aside from adipose tissue. Lipids such as cholesterol, sphingolipids, and glycerophospholipids not only serve as core structural components of neuronal cell membranes and myelin sheaths, but also participate in diverse physiological processes including signal transduction, mitochondrial function maintenance, and neuroinflammation regulation [2]. With the rapid development of lipidomics and metabolomics, breakthrough progress has been made in research on the association between lipid metabolism disorders and CNS diseases [3]. Significant abnormalities in lipid metabolism occur throughout the entire disease course in neurodegenerative diseases such as Alzheimer's disease (AD) and Parkinson's disease (PD); CNS trauma including spinal cord injury (SCI) and traumatic brain injury (TBI); as well as cerebrovascular diseases such as stroke and intracerebral hemorrhage (ICH) [4, 5, 6, 7]. After CNS injury, abnormal lipid metabolism and lipid accumulation occur in neurons, astrocytes, and other cells, triggering irreversible cellular damage and initiating pathological cascades that form a vicious cycle of disease progression [8, 9].

As of now, the incidence and disability rates of CNS diseases worldwide show a continuous upward trend [10]. Globally, the number of people living with AD has exceeded 50 million and is still rising rapidly [11]. Traumatic disorders such as SCI and TBI lead to severe neurological deficits in patients, while stroke ranks as the leading lethal neurological disease worldwide [10]. Mostly, current clinical treatments are symptomatic and supportive, lacking curative strategies targeting the core pathological mechanisms [12]. As a pivotal hub in the pathological processes of various CNS diseases, lipid metabolism, when fully elucidated for its regulatory mechanisms, can provide novel directions for targeted therapy. This review summarizes the pathological roles and molecular mechanisms of lipid metabolism disorders in different CNS diseases, as well as the clinical therapeutic strategies and research progress targeting lipid metabolism, laying a theoretical foundation for clarifying disease pathogenesis and developing specific targeted therapies.

This review focuses on AD, PD, SCI, TBI, stroke, and ICH. These six disorders were selected because they collectively represent three distinct pathophysiological paradigms in which lipid metabolism plays a central and mechanistically well‐established role: (1) protein aggregation‐driven neurodegeneration (AD and PD), (2) secondary injury cascades following mechanical trauma (SCI and TBI), and (3) vascular insufficiency and hemorrhagic injury (stroke and ICH). While other CNS conditions such as multiple sclerosis (MS) and amyotrophic lateral sclerosis (ALS) also involve lipid dysregulation, their underlying mechanisms, primarily autoimmune‐mediated demyelination and motor neuron‐specific degeneration, respectively, are distinct from the pathological frameworks addressed here [13, 14]. We aim to provide an in‐depth analysis of these representative disorders to illuminate both shared and disease‐specific lipid metabolic pathways, thereby offering a focused reference for future mechanistic and therapeutic research.

Prior reviews have generally addressed lipid dysregulation in individual CNS disorders [15, 16, 17], yet few systematically compare shared and disease‐specific lipid mechanisms across multiple pathological subtypes. To address this gap, this review focuses on six representative CNS diseases covering neurodegeneration, traumatic secondary injury, and cerebrovascular damage. We highlight conserved pathogenic lipid cascades and unique disease‐related lipid alterations, summarize updated lipid‐targeting therapeutic advances, and discuss current translational challenges. This cross‐disease comparison provides distinctive mechanistic insights and offers a focused theoretical framework for future mechanistic exploration and therapeutic development in CNS lipid research.

## 2 Literature Search Strategy

This article is a narrative review summarizing the current understanding of lipid metabolism in CNS diseases. A comprehensive literature search was conducted across PubMed, Web of Science, and Ovid databases to identify relevant studies published between 2020 and 2026. The final search was performed on March 14, 2026.

The search strategy employed a combination of keywords and Medical Subject Headings (MeSH) terms, including “lipid metabolism,” “central nervous system,” “neurodegenerative diseases,” “Alzheimer's disease,” “Parkinson's disease,” “traumatic brain injury,” “spinal cord injury,” “stroke,” and “intracerebral hemorrhage”, with selection criteria as follows: (1) original research articles and high‐impact reviews published in peer‐reviewed English‐language journals; (2) studies focusing on the pathophysiological mechanisms of lipid metabolism in the context of the six selected CNS disorders; and (3) preclinical (in vitro and in vivo) and clinical studies providing insights into lipid‐related pathways. Articles were excluded if they were not directly relevant to the core topics, were published in languages other than English, or were duplicate publications. After screening titles, abstracts, and full texts, a final selection of literature was made to ensure a focused and systematic discussion.

## 3 Lipid Metabolism in the CNS

**FIGURE 1:**
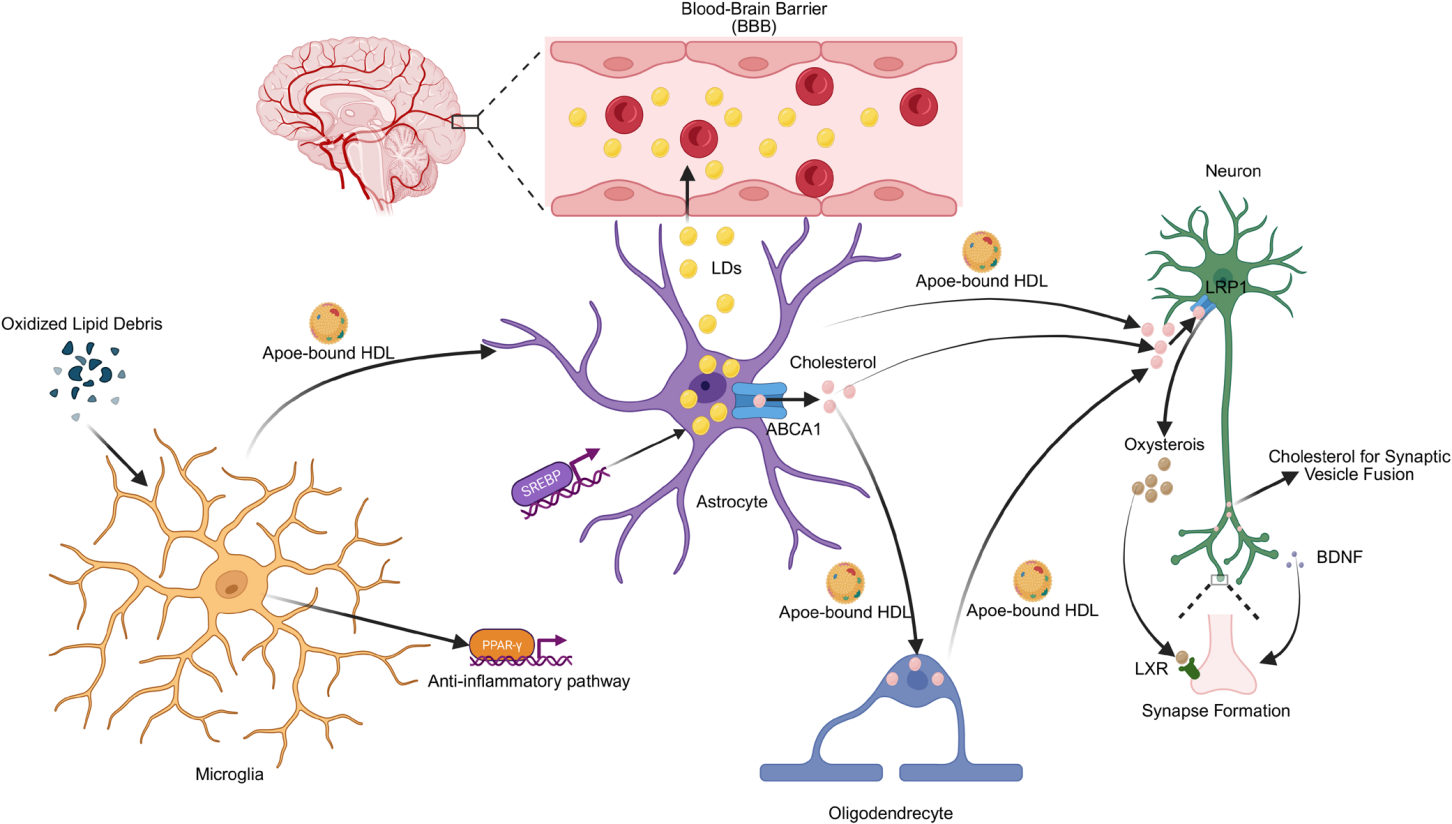
CNS lipid metabolism. The CNS forms an independent lipid metabolism system isolated by the BBB. Lipids are mainly synthesized in situ and rely on the synergistic interaction of neurons, astrocytes, oligodendrocytes, and microglia to maintain homeostasis. Astrocytes transport cholesterol to neurons and oligodendrocytes via ApoE‐associated HDL, supporting synapse formation and myelin synthesis. Cholesterol in neurons regulates synaptic vesicle fusion and signal transmission. Oligodendrocytes utilize lipids to construct myelin structures. Microglia clear oxidized lipid debris and maintain homeostasis of the CNS microenvironment. This figure was created by www.Biorender.com.

The CNS is one of the most lipid‐dense organ systems, with highly specific and complex lipid metabolism. Beyond serving as structural components of biological membranes, lipids exert key functions in sustaining neuronal signal transduction, synaptic plasticity, energy metabolism, and the modulation of neuroinflammation [18, 19, 20]. There are four main types of lipids in the CNS: glycerolipids, sphingolipids, glycerophospholipids, and fatty acids [2, 20]. Within contrast to peripheral tissues, lipid metabolism in the CNS is relatively independent. Due to the barrier effect of the blood–brain barrier (BBB), which prevents peripheral lipid influx, cholesterol and other lipids in brain tissue mainly rely on local synthesis accordingly [21]. Moreover, lipid metabolism interacts across different cell types in the CNS [22, 23, 24] (Figure 1).

### 3.1 Lipid Metabolism in Neurons

The dependence of neurons on lipid metabolism is primarily reflected in the maintenance of membrane structure and synaptic function [25]. Neuronal membranes are rich in phospholipids and cholesterol, which not only maintain membrane fluidity and integrity but also regulate the fusion of synaptic vesicles and the release of neurotransmitters [2, 26]. Although neurons possess endogenous biosynthetic machinery, they predominantly rely on lipids supplied by astrocytes, and most neuronal cholesterol originates from glial cells [27]. Cholesterol is enriched in both the presynaptic and postsynaptic membranes, regulating the efficiency and precision of synaptic transmission [28]. Neurons generate oxysterol metabolites and interact with liver X receptors (LXRs) to regulate cholesterol homeostasis and balance [29]. During development, neuronal cholesterol synthesis is crucial for the growth of axons and dendrites as well as synapse formation [30]. Brain‐derived neurotrophic factor (BDNF) upregulates cholesterol synthesis within neurons, thereby promoting synapse formation and function [29].

### 3.2 Lipid Metabolism in Astrocytes

Astrocytes represent the core cell type for lipid synthesis in the CNS, governing the biosynthesis of cholesterol and fatty acids [21, 31]. Astrocytes deliver cholesterol to neurons and oligodendrocytes via apolipoprotein E (ApoE)‐mediated vesicular transport, which is essential for maintaining neuronal function and myelination, lipid metabolism in oligodendrocytes [32, 33, 34]. Within high‐density lipoproteins (HDL), ApoE regulates intercellular cholesterol transport and membrane remodeling through receptor‐dependent endocytosis [35]. ATP‐binding cassette transporter A1 (ABCA1) expressed in astrocytes facilitates the lipidation of ApoE and the formation of HDL‐like particles, mediating cholesterol efflux and redistribution [36]. Moreover, ApoE, upon binding cholesterol, can promote cholesterol uptake and utilization in neurons through members of the low‐density lipoprotein receptor (LDLR) family, such as low‐density lipoprotein receptor‐related protein 1 (LRP1) [37]. Meanwhile, astrocytes form lipid droplets (LDs) to store excess lipids and prevent lipotoxicity, providing energy for neurons under stress conditions. The intracellular sterol regulatory element‐binding protein (SREBP) pathway directly regulates lipid synthesis and thereby affects synaptic development [38, 39].

### 3.3 Lipid Metabolism in Oligodendrocytes

Oligodendrocytes are responsible for myelin formation in the CNS, and myelin is a highly lipid‐rich structure, with lipids accounting for over 80% of its dry weight [40]. Myelin primarily contains sphingomyelin, cholesterol, and galactocerebroside, and its synthesis is highly dependent on lipid metabolic pathways [41]. Cholesterol is crucial for the compactness and function of myelin membranes, and its metabolic disorder leads to abnormal myelin development or demyelination [42]. During CNS development, the lipid synthesis capacity of oligodendrocytes is significantly enhanced, and the expression of related genes is regulated via SREBP and LXR pathways, ensuring lipid supply for myelin synthesis [43, 44, 45].

### 3.4 Lipid Metabolism in Microglia

Microglia, the resident immune cells of the CNS, play a pivotal role in lipid sensing, uptake, and metabolism [46]. Lipid fragments released from damaged tissues, such as oxidized phospholipids and free fatty acids, can be recognized and cleared by microglia, while simultaneously activating inflammatory signaling pathways [47, 48]. Lipid accumulation also affects their phenotypic transition, promoting shifts toward either pro‐inflammatory or anti‐inflammatory states [49]. Lipid metabolism is regulated via the peroxisome proliferator‐activated receptor (PPAR) signaling pathway, thereby modulating inflammatory responses [50]. In particular, the activation of PPARγ exerts anti‐inflammatory effects and inhibits the release of pro‐inflammatory factors, serving as a key target for regulating microglial function [51, 52].

## 4 Role of Lipid Metabolism in CNS Diseases

**FIGURE 2:**
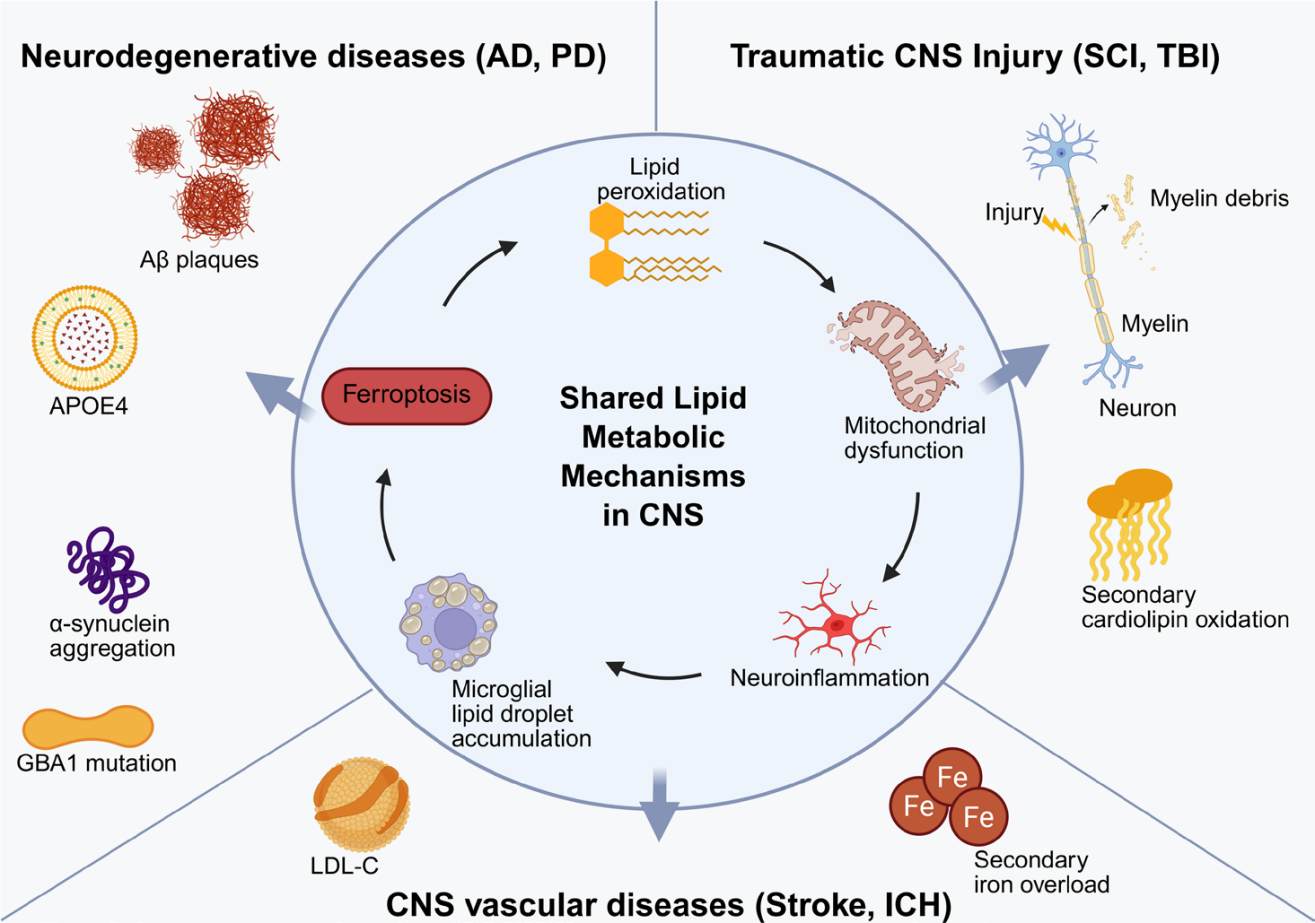
Schematic diagram of shared and disease‐specific lipid‐related mechanisms in CNS disorders. These six diseases share common pathological cascades, including microglial lipid droplet accumulation, neuroinflammation, lipid peroxidation, mitochondrial dysfunction, and ferroptosis. Vascular diseases (stroke and ICH) are triggered by vascular insufficiency and hematoma formation, leading to downstream iron overload; neurodegenerative diseases (AD and PD) arise from protein aggregation and genetic susceptibility; traumatic CNS injuries (SCI and TBI) are initiated by mechanical impact, followed by secondary lipid peroxidation. All disease‐specific pathways eventually converge into the shared central pathological vicious cycle. This figure was created by www.Biorender.com.

This review summarizes the pathological roles and molecular mechanisms of lipid metabolism disorders in AD, PD, SCI, TBI, stroke, and ICH. We identify several shared pathogenic cascades, including lipid peroxidation, ferroptosis, neuroinflammation, mitochondrial dysfunction, and microglial lipid droplet accumulation, while also outlining the disease‐specific triggers for each disorder (Figure 2).

## 5 Role of Lipid Metabolism in Neurodegenerative Diseases

Lipids play important roles in various cell types [21]. The brain is highly vulnerable to disrupted lipid homeostasis, which is crucial for the development and progression of neurodegenerative diseases and serves as a vital regulator in pathological processes [53, 54].

Neurodegenerative diseases are pathologically characterized by progressive neuronal loss and abnormal protein deposition, among which AD and PD are the two most prevalent representative disorders [55]. Lipidomics and metabolomics studies have confirmed that dysregulated lipid metabolism is one of the key mechanisms driving the pathogenesis and progression of AD and PD [56, 57]. Imbalanced lipid homeostasis significantly increases the risk of AD and PD, mainly manifested as pathological protein aggregation induced by abnormal lipid levels and oxidative stress injury mediated by lipid peroxidation. These metabolic alterations markedly deviate from normal physiological status [58, 59]. Furthermore, lipid metabolic abnormalities can continuously promote disease development and deterioration by regulating multiple signaling pathways, including pathological protein aggregation, mitochondrial dysfunction, and neuroinflammation [25, 60].

### 5.1 Lipid Metabolism Disruption in AD

AD is a typical neurodegenerative disorder, accounting for approximately 60% to 70% of dementia cases worldwide [10]. It is clinically characterized by a progressive decline in cognitive function. The major pathological features include the deposition of β‐amyloid (Aβ) plaques, hyperphosphorylation of tau protein, and the formation of neurofibrillary tangles, accompanied by pathological alterations such as neuronal and synaptic damage and loss, chronic neuroinflammation, and brain atrophy [61, 62, 63]. Among these, the accumulation of abnormal tau protein and Aβ represents the most central hallmark of AD [64].

As a highly lipid‐rich organ, the brain relies on the homeostasis of lipid metabolism to maintain the structural integrity and normal physiological functions of neuronal cells [65, 66]. Once lipid homeostasis is disrupted, a cascade of downstream pathological events will be triggered, thereby aggravating the progression of AD [67, 68]. Accumulating evidence suggests that aberrant lipid metabolism serves as a critical hub regulating multiple pathological changes in AD [56]. Its dysregulation persists throughout the entire disease course and is closely associated with key pathological events such as Aβ deposition, tau abnormalities, and neuroinflammation [69]. Studies have confirmed that the accumulation of lipids such as cholesterol and sphingolipids is closely linked to the abnormal aggregation of Aβ and tau proteins, jointly participating in the pathological process of AD [70, 71].

The human brain contains 25% of total body cholesterol [72]. Owing to the barrier function of the BBB, the vast majority of cholesterol in the brain is synthesized within the CNS [73]. Cholesterol levels are significantly elevated in the periphery and brain of AD patients, with abnormal cholesterol accumulation in senile plaque regions that are positively correlated with disease severity [74, 75, 76, 77].

Excessive cholesterol accumulation in the CNS microenvironment further upregulates γ‐secretase activity, aggravates abnormal cleavage of amyloid precursor protein (APP), increases Aβ generation and deposition, and drives microglial polarization toward a pro‐inflammatory phenotype [78, 79, 80]. This process releases inflammatory factors, forming a “lipid accumulation‐Aβ deposition‐neuroinflammation” cascade that continuously exacerbates neuronal damage [81]. Additionally, high cholesterol levels in neurons enhance Aβ‐induced oxidative stress in mitochondria, inhibit the fusion of damaged mitochondria with lysosomes, causing the accumulation of dysfunctional mitochondria, ultimately leading to neuronal death [82, 83, 84].

The *APOE* gene is the most important genetic risk gene for AD, and one of its core physiological functions is to mediate the transport and metabolism of cholesterol in the brain, a function closely related to the pathological occurrence of AD [85]. The APOE4 allele in the general population is a strong risk factor for late‐onset Alzheimer's disease (LOAD), which markedly increases disease susceptibility, advances the age of onset, and mediates abnormal intracellular cholesterol accumulation [17, 86, 87]. Abnormalities in the TREM2‐mediated ApoE pathway disrupt brain microenvironmental homeostasis, create a positive feedback loop with lipid metabolic disorders, and aggravate neuronal degeneration [88]. Cerebrovascular and BBB damage further exacerbates abnormal lipid metabolism and promotes the pathological progression of AD [89] (Figure S1).

### 5.2 Lipid Metabolism Disruption in PD

PD is the second most common neurodegenerative disease after AD, characterized by progressive loss of dopaminergic neurons in the substantia nigra and abnormal aggregation of α‐synuclein (α‐Syn) [90, 91]. Dysregulated lipid metabolism is deeply involved in the pathological progression of PD. It aggravates mitochondrial dysfunction, oxidative stress injury, and iron metabolic imbalance, thereby triggering chronic neuroinflammation [92, 93, 94]. Meanwhile, it activates microglia and promotes the release of pro‐inflammatory cytokines, forming a cascade of pathological reactions that accelerate the degeneration and apoptosis of dopaminergic neurons [95].

At the genetic level, abnormalities in lipid metabolism genes are closely associated with the risk of PD, among which glucosylceramidase beta 1 (GBA1) mutation represents a major genetic risk factor for PD. The mutation reduces the activity of β‐glucocerebrosidase (GCase), leading to lysosomal dysfunction and subsequent impairment of brain metabolism, ultimately resulting in abnormal accumulation of cholesterol and polyunsaturated fatty acids (PUFAs) [70]. Increased levels of PUFAs upregulate the expression of α‐Syn synthesis genes, aggravate the abnormal production and aggregation of α‐Syn, thus impairing the structure and function of dopaminergic neurons, which significantly increases the risk of PD [96, 97].

Moreover, astrocytes generate excessive LDs in the brain, transporting toxic fatty acids to neurons through ApoE‐mediated transport process, further aggravating neuronal damage [98]. In addition, abnormalities of fatty acid metabolism‐related enzymes are also involved in PD progression. For example, aberrant activation of acyl‐CoA synthetase long‐chain family member 4 (ACSL4) promotes lipid peroxidation, inducing neuronal ferroptosis, accelerating the loss of dopaminergic neurons [99].

Phospholipids are the main components of cellular biological membranes, and sphingolipids, as an important class of phospholipids, act as precursors of lipid signaling molecules to participate in cellular signal transduction [100]. Calcium‐independent phospholipase A_2_ (PLA_2_) is one of the key pathogenic factors in PD [92, 101, 102]. PLA_2_ plays a crucial role in the maintenance and survival of dopaminergic neurons, as well as in the stability of α‐Syn [103, 104]. In mouse models of PD, overexpression of mutant A53T α‐Syn downregulates PLA_2_ expression, thereby promoting the accumulation of oxidized phospholipids and ultimately inducing ferroptosis [105]. Conversely, loss of PLA_2_ gene function also leads to lipid peroxidation and mitochondrial dysfunction [106] (Figure S2).

## 6 Role of Lipid Metabolism in Traumatic CNS Injury

Traumatic CNS injury mainly includes TBI and SCI. The pathophysiological process of TBI and SCI can be divided into primary injury and secondary injury [107, 108]. Primary injury is directly induced by mechanical force, characterized by tissue tearing and acute neuronal necrosis. Secondary injury is a cascade reaction triggered by multiple factors, including inflammation, oxidative stress, and lipid metabolism dysregulation, leading to the continuous deterioration of neurological function after injury [109]. Lipid metabolism dysregulation not only plays a key role in the occurrence and development of secondary injury but also participates in regulating nerve repair and tissue remodeling after injury.

### 6.1 Lipid Metabolism Disruption in SCI

SCI is a severe traumatic disease of the CNS that often results in permanent motor and sensory dysfunction in patients. Following primary injury, a cascade of complex secondary pathological responses is triggered, mainly including neuroinflammation, neuronal apoptosis, myelin damage, and glial scar formation [110, 111].

Dysregulation of lipid metabolism serves as a regulatory factor underlying these pathological processes [112, 113]. It not only directly participates in early neuronal death but also triggers cascaded pathological responses such as neuroinflammation and demyelination by regulating microglial activation, thereby aggravating neurological damage after SCI. Among the processes, ferroptosis driven by iron‐dependent lipid peroxidation represents an important pathway of neuronal injury following SCI [5]. Notably, abnormal activation of microglia, which is closely associated with disturbed lipid metabolism, further amplifies the impact after SCI, ultimately leading to neuronal damage and neurodegenerative changes [114, 115].

After SCI, a large amount of myelin debris accumulates in the injured region, directly inhibiting axonal and myelin regeneration and mediating persistent demyelination [116, 117]. Microglia act as scavengers of myelin debris; their phagocytosis and metabolic clearance of lipid‐rich myelin debris are critical for initiating tissue repair and restricting inflammatory spread [118]. However, the high cholesterol content in myelin debris often exceeds the lipid efflux capacity of phagocytes, resulting in abnormal intracellular cholesterol accumulation [119] and the formation of lipid droplet‐ and cholesterol crystal‐laden foam‐like microglia (LDAM) [49]. This cluster of microglia exhibit marked phagocytic dysfunction, generate abundant ROS, and secrete various pro‐inflammatory cytokines, thereby further amplifying local tissue damage [49, 120]. In addition, excessive cholesterol accumulation in microglia also promote glial scar formation, which severely impairs neurological functional recovery [113].

Macrophages, which are homologous to microglia, also exhibit similar lipid regulatory functions after SCI. Because of the disruption of the BBB caused by SCI, macrophages infiltrating the lesion core phagocytose large amounts of myelin debris [121]. However, impaired autophagy or dysfunctional lipid efflux pathways lead to continuous intracellular accumulation of cholesterol esters and neutral lipids, driving macrophage transformation into foam cells. These cells persistently release pro‐inflammatory cytokines and exacerbate tissue damage [122].

As a key transporter regulating lipid metabolism, ABCA1 plays a crucial role in the removal of myelin debris [123]. ABCA1 primarily mediates the efflux of intracellular cholesterol and phospholipids to extracellular lipoproteins [124]. Upregulation of ABCA1 expression enhances lipid efflux efficiency and inhibits foam cell formation [125], while indirectly suppressing the activation of intranuclear inflammatory signaling pathways and alleviating inflammatory cascades and subsequent neurological injury [116].

Furthermore, dysregulated lipid metabolism in macrophages after SCI is closely correlated with oxidative stress and lipid peroxidation damage. The markedly elevated level of 4‐Hydroxy‐2‐nonenal (4‐HNE) in injured tissues further confirms the pivotal role of lipid peroxidation in the pathological progression of SCI [126, 127, 128, 129].

Overall, lipid metabolism disorder serves as a vital driver of secondary pathological injury following SCI. It exacerbates neural tissue damage and impairs functional recovery by regulating multiple key pathways, including impaired myelin clearance, foam cell formation, chronic neuroinflammation, oxidative stress, and glial scar formation (Figure S3).

### 6.2 Lipid Metabolism Disruption in TBI

TBI is a type of CNS injury that causes severe cognitive and motor function disorders. This kind of injury not only involves the primary structural damage directly mediated by mechanical force, but also triggers a series of complex and continuous secondary biochemical cascade reactions [130]. The disruption of lipid homeostasis has been confirmed as one of the mechanisms driving the progression of neurodegenerative changes after injury [53].

TBI is pathologically characterized in the early stage by abnormal degradation of cell membrane phospholipids and accumulation of toxic metabolites [131]. Trauma‐induced oxidative stress can activate cytosolic phospholipase A_2_ (cPLA_2_) [132], which specifically degrades glycerophospholipids such as PC and PE in the brain and generates intermediate products including lysophospholipids (LPLs) and free fatty acids [131, 133]. The abnormal accumulation of lysophosphatidylcholine (Lyso‐PC) in the brain directly induces neuronal apoptosis, axonal rupture, and synaptic dysfunction [134]. Reactive aldehydes such as 4‐HNE produced by lipid peroxidation can modify proteins and nucleic acids, amplify oxidative damage, and form a vicious cycle of oxidative damage and lipid degradation, thereby driving the early pathological progression of TBI [135, 136].

Mitochondrial lipid‐specific oxidative injury is a key inducer of cellular energy metabolism disturbance following TBI [137, 138]. As a characteristic phospholipid in the inner mitochondrial membrane, cardiolipin is essential for maintaining the structural and functional integrity of the respiratory chain and is highly vulnerable to oxidative stress attack [139, 140]. After TBI, the content of mitochondrial cardiolipin in the brain decreases and undergoes severe peroxidation modification, which directly inhibits respiratory chain electron transport, reduces ATP production, triggers collapse of the mitochondrial membrane potential and the release of apoptotic factors, and disrupts mitochondrial quality control pathways including fission, fusion, and mitophagy [132, 141, 142]. In the elderly population, the capacity for mitochondrial lipid repair declines markedly after TBI, leading to an imbalance between pro‐inflammatory fatty acids such as arachidonic acid and anti‐inflammatory fatty acids such as DHA. This persistently activates microglia and astrocytes, ultimately exacerbating neuroinflammation and neurological deficits [132, 133, 143].

In addition, lipid metabolic reprogramming and abnormal accumulation of LDs also participate in the regulation and evolution of neuroinflammation after TBI [144, 145]. Reversible acetylation of fatty acid synthase (FASN) in microglia is induced after TBI, thereby up‐regulating the lipid synthesis pathway and driving the accumulation of LDs in microglia [146]. By altering the phenotype of microglia, the release of proinflammatory cytokines is increased, thereby exacerbating inflammatory damage to the CNS (Figure S4).

In addition, TBI disrupts hypothalamic regulation of systemic lipid metabolism via the brain–body axis, resulting in hepatic lipid accumulation and reduced insulin sensitivity. Peripheral metabolic disturbance, in turn, exacerbates the pathological processes in the brain [147, 148].

## 7 Role of Lipid Metabolism in CNS Vascular Diseases

Vascular diseases of the CNS mainly include stroke and ICH, characterized by abnormal changes in the structure and function of cerebral blood vessels. Dysregulation of lipid metabolism is a crucial risk factor for the development and progression of these diseases. It is widely involved in the pathogenesis by modulating key pathological processes including atherosclerosis, vascular endothelial dysfunction, and thrombosis [149, 150].

### 7.1 Lipid Metabolism Disruption in Stroke

Stroke is one of the leading neurological diseases with the highest disability and mortality rates worldwide [151, 152]. Lipid metabolism disruption is not only involved in the regulation of stroke risk factors but also occurs throughout acute brain injury, neuroinflammatory cascades, and chronic neurological functional remodeling [153].

In the pathological process of ischemic stroke, abnormal metabolism of low‐density lipoprotein cholesterol (LDL‐C) is a key event, and its dysregulation is closely associated with stroke risk and disease severity [154, 155]. Under physiological conditions, LDL‐C acts as the major transporter of cholesterol and participates in systemic lipid metabolism [156]. A large prospective cohort study involving 267,500 individuals from the general Chinese population confirmed a significant positive association between LDL‐C levels and the risk of ischemic stroke. After adjusting for confounding factors, each 1 mmol/L increase in serum LDL‐C concentration was associated with an 8% higher risk of ischemic stroke in the population [157]. Dysregulated LDL‐C metabolism predisposes to its deposition in the vascular endothelium, which activates inflammation and promotes monocyte infiltration and differentiation into macrophages. Macrophages take up excessive lipids and transform into foam cells, driving the formation and growth of lipid plaques and consequently leading to cerebral vascular stenosis and occlusion [158, 159]. Plaque rupture can trigger thrombosis, thereby forming a pathological vicious cycle of lipid deposition—inflammatory response—plaque formation—thrombotic occlusion [160].

In addition, the hypoxic microenvironment in the brain after ischemic stroke induces high expression of HIF‐1α [161]. HIF‐1α activates the fatty acid synthesis pathway and inhibits fatty acid oxidation, resulting in lipid accumulation and energy metabolic disorder in glial cells, which aggravates neuronal apoptosis [162, 163]. Its regulatory effect on lipid metabolism exhibits spatiotemporal specificity, providing a time‐window target for targeted therapy [153, 164](Figure S5).

### 7.2 Lipid Metabolism Disruption in ICH

ICH is highly disabling and fatal [165]. Lipid metabolism is highly specific in the risk assessment of ICH. It is deeply involved in secondary brain injury and the regulation of neural function repair after hematoma formation [166, 167]. Lipid peroxidation is a key driver of neuronal death following ICH. After ICH, substances such as iron ions released from the hematoma catalyze the production of large amounts of ROS, thereby triggering intense lipid peroxidation of cell membranes [168].

Following ICH, fatty acid‐binding protein 4 (FABP4) is markedly upregulated in microglia. It not only exacerbates pathological lipid droplet accumulation, but also promotes microglial polarization toward the pro‐inflammatory M1 phenotype by inhibiting the degradation of the pro‐inflammatory protein S100A9. This process induces the secretion of neurotoxic factors and recruits neutrophil infiltration, further amplifying neuroinflammation and aggravating pathological damage after ICH [166] (Figure S5).

## 8 Treatment Targeting Lipid Metabolism

At present, a variety of lipid‐targeted interventions are available for different CNS diseases, yet they differ drastically in research and development stages. All these therapeutic approaches generally suffer from poor BBB penetration, insufficient target specificity, and prominent obstacles to clinical translation. Relevant intervention strategies and their mechanisms will be elaborated by disease type in the following sections (Table 2).

### 8.1 AD Treatment Targeting Lipid Metabolism

PC is the most abundant phospholipid in the presynaptic membrane, and insufficient synthesis of PC impairs synaptic structure and disrupts neurotransmitter release. Supplementary CDP‐choline can cross the BBB, convert into PC and cytidine to repair neural membranes, and activate the PI3K pathway to inhibit neuronal apoptosis. Long‐term administration improves cognitive function and reduces Aβ levels in AD patients, a mechanism associated with the inhibition of γ‐secretase activity [169].

Brain cholesterol homeostasis relies on the balance between synthesis and degradation. Statins inhibit the key synthetic enzyme HMG‐CoA reductase and upregulate ApoE expression to mediate cholesterol transport and facilitate Aβ clearance [170]. Gene editing targeting the APOE4 genotype to upregulate APOE2 has entered the preclinical stage [171]. In addition, the key enzyme of cholesterol clearance in the brain, CYP46A1, can convert cholesterol into metabolites that can cross the BBB [36].

Patients with AD exhibit an imbalance of sphingolipid metabolism in the brain, characterized by downregulation of GM1 and sphingosine accumulation, which exacerbates neurodegeneration. GM1 can bind to Aβ and inhibit its aggregation, whereas sphingosine accumulation activates apoptotic pathways and accelerates neuronal death [172, 173]. Exogenous supplementation of GM1 improves synaptic plasticity in patients with mild AD; however, its low permeability across the BBB limits clinical application [172, 174].

### 8.2 PD Treatment Targeting Lipid Metabolism

Lipid metabolism dysregulation in PD is mainly manifested as abnormal binding of α‐Syn to lipids in the brain and enhanced apoptosis mediated by ceramide [175, 176]. Treatment strategies are mainly aimed at protecting dopaminergic neurons.

The metabolism of ceramide in the substantia nigra pars compacta of PD reveals complex abnormal changes. One of its mechanisms is the pathological activation of ceramide synthase, which leads to the accumulation of ceramide in dopamine neurons and then initiates programmed neuronal death by activating caspase cascade [177, 178]. In the aspect of targeted interventions, CERs‐specific inhibitors, such as Fumonisin B1, can block the *de novo* synthesis of ceramide, significantly reduce ceramide accumulation in PD animal models, and increase the survival rate of dopamine neurons [179].

### 8.3 SCI Treatment Targeting Lipid Metabolism

Lipid metabolism is crucial for secondary injury, when phospholipid degradation produces a large number of inflammatory mediators, myelin lipid loss leads to axonal demyelination, and abnormal cholesterol metabolism inhibits nerve repair [180].

After SCI, rapid PLA_2_ overactivation degrades membrane phospholipids [181]. The released AA generates pro‐inflammatory PGE_2_ via the COX pathway and exacerbates local inflammation [182]. PLA_2_ inhibitors suppress AA release, boost anti‐inflammatory resolvins, and alleviate glial infiltration [183]. The LOX inhibitor zileuton blocks AA metabolism, reduces leukotriene production, and synergizes with resolvins to improve motor function [184, 185]. Elevated lysophosphatidic acid (LPA) mediates demyelination through LPA_1_, and the LPA_1_ antagonist AM095 effectively mitigates demyelinating lesions [186].

Oligodendrocyte apoptosis triggers myelin destruction, leading to neurological dysfunction [187, 188]. Galactocerebroside provides precursors for myelin regeneration [189]. ABCA1 mediates cholesterol transport to supply raw materials for myelin synthesis; upregulation of its expression promotes myelin and axonal repair and facilitates the recovery of neurological function [190, 191].

### 8.4 TBI Treatment Targeting Lipid Metabolism

Mitochondrial dysfunction and energy metabolism remodeling are classic pathological features of TBI. Oxidative stress injury to cardiolipin acts as a crucial initiator that triggers subsequent cascading pathological damage [132]. Traditional nonspecific global antioxidants have mostly failed in the treatment of TBI [128]. In contrast, mitochondria‐targeted electron scavengers can accumulate in brain mitochondria, inhibit cardiolipin oxidation, protect endogenous antioxidant substances in the brain, and thereby markedly improve neurological function [137].

Ferroptosis is driven by lipid peroxidation and iron overload, which is rapidly activated after TBI, accompanied by the upregulation of ACSL4 and Arachidonate 15‐lipoxygenase (ALOX15) as well as GSH depletion [5]. Ferroptosis inhibitors such as Ferrostatin‐1 and Liproxstatin‐1 restrain lipid disorders and reduce iron accumulation, thereby exerting neuroprotective effects [192, 193, 194]. Inhibitors of PLA_2_, as well as CDP‐choline and phospholipase C inhibitors, regulate the lipid peroxidation cascade and alleviate inflammation and brain injury [195, 196, 197].

High expression of lipoprotein lipase (LPL) in microglia exacerbates inflammation. Orlistat inhibits its abnormal activation, regulates lipid metabolic pathways, and promotes microglial polarization toward the anti‐inflammatory M2 phenotype [198]. TREM2 facilitates cholesterol efflux and recycling via the 24‐dehydrocholesterol reductase (DHCR24)/LXR pathway, accelerates the differentiation of oligodendrocyte precursor cells, alleviates white matter damage, and promotes functional recovery [199].

Furthermore, multiple antioxidant and lipid supplementation therapies can alleviate lipid peroxidation. Vitamin E scavenges lipid peroxyl radicals and suppresses lipid peroxidation. Preclinical studies have demonstrated its beneficial effects in TBI [200, 201]. Vitamin C levels in the brain decrease following TBI [202], and high‐dose administration can reduce cerebral edema [203]. N‐acetylcysteine (NAC), γ‐glutamylcysteine ethyl ester, and other compounds can replenish GSH and improve TBI‐related outcomes. Other antioxidants such as Curcumin and Resveratrol have shown varying degrees of neuroprotective potential in experimental studies [204, 205].

### 8.5 ICH and Stroke Treatment Targeting Lipid Metabolism

Currently, clinical treatments for ICH are limited and therapeutically unsatisfactory. Ferroptosis and lipid metabolism disorders represent highly promising therapeutic targets [206, 207, 208]. Ferrostatin‐1, LOX inhibitor 1, and Compound 51 can exert neuroprotective effects by inhibiting lipid peroxidation and regulating ferroptosis [209, 210, 211]. Antioxidant agents such as Resveratrol, Nanoformulations, and NAC can scavenge ROS, reduce lipid peroxides, and alleviate secondary brain injury. Among them, NAC has entered clinical trials [212, 213]. The iron chelator Deferoxamine reduces iron accumulation and inhibits PUFA lipid peroxidation, thereby improving both early and long‐term neurological functional outcomes in patients with ICH [214, 215].

The process of neuroinflammation after ICH is closely related to and interacts with cholesterol disorders. Maintaining cholesterol homeostasis alleviates CNS injury and promotes neurological functional recovery [216]. The LXR signaling pathway can simultaneously inhibit inflammatory responses, promote cholesterol transport and recycling, and thereby support remyelination. Its agonists have exhibited significant neuroprotective potential in relevant models [217, 218, 219].

Lipid‐targeted therapy for stroke mainly focuses on regulating lipid metabolism, exerting anti‐inflammatory effects, and protecting vascular endothelium. Statins remain the first‐line medications for ischemic stroke, and PCSK9 inhibitors can further reduce low‐density lipoprotein levels and lower the risk of stroke recurrence [220, 221, 222]. Lipid mediator modulators such as LOX inhibitors can suppress the production of pro‐inflammatory Hydroxyeicosatetraenoic acids (HETEs), promote the synthesis of Resolvins, alleviate neuroinflammation, and improve neurological recovery after ischemic stroke [223, 224, 225]. COX‐2 inhibitors can precisely regulate PGE_2_ levels, exerting neuroprotective effects in the acute phase and preventing exacerbated inflammation caused by high concentrations of PGE_2_ in the chronic phase [226].

Persistent cholesterol accumulation after stroke continuously activates microglia, while CYP46A1 mediates cholesterol metabolism and clearance [227, 228]. Low‐dose efavirenz can cross the BBB to activate CYP46A1, promote cholesterol efflux, inhibit inflammation, and facilitate neurological functional recovery [158].

## 9 Emerging Omics Technologies for Deciphering Lipid Metabolic Alterations in CNS Diseases

The rapid advancement of lipidomics and other high‐throughput omics technologies has enabled the precise dissection of CNS lipid metabolic disorders at cell‐type, regional, and even subcellular resolution [229]. Large‐scale bulk lipidomics (LC–MS/MS) has been widely applied to quantify lipid species in brain tissues and biofluids, revealing global lipid signatures in AD, PD, SCI, TBI, stroke, and ICH—for example, elevated cholesteryl esters and ceramides in AD, increased cardiolipin oxidation in TBI, and altered glycerophospholipid profiles in ischemic stroke [230, 231, 232]. Spatial lipidomics, exemplified by matrix‐assisted laser desorption/ionization mass spectrometry imaging (MALDI‐MSI), enables direct mapping of lipid distribution across tissue sections and links region‐specific lipid alterations to pathological features. For instance, focal sulfatide accumulation is observed surrounding amyloid plaques in AD [233].

Single‐cell/single‐nucleus transcriptomics coupled with single‐cell lipidomics identifies cell‐type‐specific lipid metabolic disorders in CNS diseases: dopaminergic neurons and glia show divergent sphingolipid and fatty acid metabolism in PD [234]; foam cells trigger persistent inflammation after SCI and TBI [235, 236]. Spatial transcriptomics platforms map HIF‐1α‐dependent spatiotemporal activation of lipid synthesis linked to neuronal damage in ischemic stroke, and integrated analysis with spatial lipidomics further uncovers underlying pathological mechanisms [153, 237].

On this basis, proteomics and metabolomics serve as important complements to lipidomics, refining the regulatory network of disrupted lipid metabolism in CNS diseases [229]. Proteomics identifies aberrant expression of lipid metabolism‐related enzymes, transporters, and regulatory proteins such as ApoE, ABCA1, and ACSL4, thereby elucidating the molecular mechanisms underlying lipid disorders [238, 239]. Metabolomics enables the detection of lipid peroxidation products including 4‐HNE and malondialdehyde, as well as antioxidant intermediates, which reflect oxidative stress triggered by lipid imbalance [240]. The multiomics strategy integrating transcriptomics, lipidomics, proteomics, and metabolomics effectively overcomes the limitations of single‐omics research. It also clarifies the crosstalk between lipid metabolism and neuroinflammation, oxidative stress, and programmed cell death, and systematically deciphers the complex pathogenic mechanisms of CNS diseases [241].

## 10 Discussion

This article reviews the regulatory role of lipid metabolism in neurodegenerative diseases, CNS trauma and cerebrovascular diseases, as well as the therapeutic prospects of targeting lipid metabolism (Tables 1 and 2). Imbalanced lipid homeostasis is not a secondary change following CNS injury, but a common important driving factor of various CNS disorders. It can amplify pathological cascades including abnormal protein aggregation, neuroinflammation, mitochondrial dysfunction, and demyelination.

**TABLE 1:** Therapeutic potential of interventions targeting lipid metabolism in CNS diseases.

Diseases	Therapeutic targets	Interventions	References
AD	PC synthesis	CDP‐choline	Agut et al. [169].
	Cholesterol synthesis	Statins	Olmastroni et al. [170].
	Cholesterol transport	APOE2 upregulation via gene editing	Golden et al. [171].
	Cholesterol clearance	CYP46A1 activation	Zhang et al. [36].
	Sphingolipid metabolism	Exogenous GM1 supplementation	Sipione et al. [172]; Nj et al. [173]; Chiricozzi et al. [174].
PD	Inhibiting the abnormal activation of CerS	Fumonisin B1	Merrill et al. [179].
SCI	PLA_2_	PLA_2_‐specific inhibitor	Li et al. [181], Liu et al. [183].
	LOX	Zileuton	Dahlke et al. [184]; Genovese et al. [185].
	LPAR1	LPAR1 antagonist, AM095	Santos‐Nogueira et al. [186].
	Cholesterol transport	ABCA1 overexpression	Oram et al. [190]; Voskuhl et al. [191].
TBI	Mitochondrial cardiolipin oxidation	Mitochondria‐targeted electron scavenger	Ji et al. [137].
	Ferroptosis (ACSL4/ALOX15 pathway)	Ferroptosis inhibitors, Ferrostatin‐1 and Liproxstatin‐1	Rui et al. [192]; Bs et al. [193]; Wu et al. [194].
	Regulating the lipid peroxidation	CDP‐choline, Phospholipase C	Adibhatla et al. [195]; Grieb et al. [196]; Golding et al. [197].
	Lipoprotein lipase	Orlistat	Yu et al. [198].
	TREM2/DHCR24/LXR pathway	TREM2 regulation	Li et al. [199].
	Lipid peroxidation	Vitamin E, Vitamin C, NAC and Resveratrol	Clifton et al. [200]; Frei et al. [202]; Lin et al. [204]; Wu et al. [205].
Stroke	LDL	Statins, PCSK9 inhibitors	Manktelow et al. [220]; Amarenco et al. [221]; Sabatine et al. [222].
	LOX	LOX inhibitor	Liu et al. [223]; Yuan et al. [224]; Rai et al. [225].
	COX‐2	COX‐2 inhibitor	Nakayama et al. [226].
	Enhancing cholesterol efflux	Efavirenz	Zhao et al. [158]
ICH	Ferroptosis/Lipid peroxidation	Ferrostatin‐1, LOX/ALOX inhibitor, Compound 51	Kajarabille et al. [209]; Wang et al. [210]; Wang et al. [211].
	Oxidative stress	Resveratrol Nanoformulation, NAC	Karuppagounder et al. [212]; Mo et al. [213].
	Iron accumulation	Deferoxamine	Guo et al. [214]; Selim et al. [215].
	Cholesterol metabolism	LXR agonist	Berghoff et al. [217]; Petrov et al. [218]; Eskandari et al. [219].

**TABLE 2:** Evidence level, model systems, disease stage, CNS delivery, clinical status, and major limitations of lipid metabolism‐targeted interventions in CNS diseases.

Intervention	Target disease	Evidence level and source	Model system	Disease stage	CNS delivery	Clinical progress and efficacy reliability	Major limitations
Statins	AD, Stroke	Clinical; From clinical trials	Human subjects	Prevention/Chronic management	Oral, crosses BBB to limited extent	Approved for secondary stroke prevention; its neuroprotective efficacy in AD lacks consistent confirmation	Low BBB penetration; controversial central neuroprotective efficacy, without consistent randomized clinical trial evidence
PCSK9 inhibitors	Stroke	Clinical; From clinical trials	Human subjects	Acute/Secondary prevention	Monoclonal antibody, does not cross BBB	Clinically validated for secondary stroke prevention; neuroprotection depends only on peripheral lipid lowering, lacking direct central efficacy evidence.	Lacks direct central effects; efficacy mainly relies on peripheral lipid regulation. Neuroprotective benefits in individual stroke patients remain unconfirmed.
CDP‐choline	AD, TBI	Clinical; From preclinical studies and small‐scale exploratory clinical trials	Human subjects	Acute (TBI)/Chronic (AD)	Oral/enteral, crosses BBB	Approved for neurological disorders in some regions; multiple clinical trials show modest and inconsistent efficacy	High heterogeneity across trial designs; modest overall neuroprotective effects; long‐term effects unconfirmed
GM1 ganglioside	AD	Clinical; From preclinical studies and small‐scale exploratory clinical trials	Mainly animal models; small‐scale exploratory clinical studies	Chronic	Poor BBB penetration	Under investigation; mild improvement signals observed in small‐scale clinical trials with inadequate evidence	Low BBB permeability; no large‐sample randomized clinical trials confirm cognitive improvement, and clinical application is limited
Ferroptosis inhibitors	SCI, TBI, ICH	Preclinical; From cellular and animal studies	Cell culture and rodent animal models	Acute/Early secondary injury	Systemic	Completed cellular and animal studies, with no human clinical trials conducted	Complete absence of clinical translation data; pharmacokinetics require optimization; risk of off‐target redox interference exists
LOX inhibitors	SCI, Stroke	Preclinical; From cellular and animal studies	Rodent models of SCI and stroke	Acute/Subacute	Oral	Limited to animal studies, no human clinical trials for CNS diseases have been conducted.	Central efficacy in humans unvalidated; potential systemic toxicity risks; long path toward clinical translation
PLA_2_ inhibitors	SCI, TBI	Preclinical; From cellular and animal studies	Rodent models for SCI and TBI	Acute	Systemic	No human trial data, only preclinical research	Insufficient subtype specificity of the target; lacking human safety data
LPA_1_ antagonist	SCI	Preclinical; From cellular and animal studies	Rodent models of SCI	Acute/Subacute	Systemic	Early animal screenin, preclinical‐to‐clinical transition not achieved	Safety in experimental animals unclear; long path to clinical translation
ABCA1 upregulators	SCI	Preclinical; From cellular and animal studies	Rodent models and in vitro microglial experiments	Subacute/Chronic	Various	Limited to basic mechanistic research with no clinical data	Optimal intervention time window undefined; human druggability unconfirmed
Mitochondria‐targeted antioxidants	TBI	Preclinical; From cellular and animal studies	Rodent models of brain injury	Acute	Targeted delivery	No human clinical trials; animal studies only	Targeted delivery to the brain is challenging; human efficacy data are lacking; major barriers exist for clinical translation
Vitamin E	TBI	Preclinical/Clinical (mixed); From preclinical studies and small‐scale exploratory clinical trials	Rodent models	Acute/Chronic	Oral	TBI neuroprotection seen solely in animal studies, clinical trial outcomes inconsistent	Clinical trial conclusions are highly divergent with marked heterogeneity
Vitamin C (high‐dose)	TBI	Preclinical/Clinical (limited); From preclinical studies and small‐scale exploratory clinical trials	Rodent models and small single‐center clinical trials	Acute	i.v./oral	Small exploratory studies suggest potential cerebral edema reduction, without large‐sample validation	Lacking large‐scale clinical evidence; neuroprotective efficacy unconfirmed
N‐acetylcysteine (NAC)	ICH, Stroke	Clinical; From preclinical studies and small‐scale exploratory clinical trials	Animal models and phase II exploratory studies	Acute	i.v./oral	In phase II exploratory trials; therapeutic signals are observed in partial groups, while overall efficacy remains unconfirmed	Unstandardized dosing regimens across disease stages; modest overall therapeutic improvement; lacking large‐scale clinical data
Deferoxamine	ICH	Clinical; From preclinical studies and small‐scale exploratory clinical trials	Human ICH patients	Acute	i.v.	Investigational neuroprotective agent for ICH; Phase II randomized trial completed, with marked patient response heterogeneity and inconsistent efficacy	High heterogeneity in patient responses; narrow therapeutic window; consistent improvement in functional outcomes unconfirmed
Curcumin	TBI	Preclinical; From cellular and animal studies	Rodent brain injury models and in vitro astrocyte/microglia experiments	Acute/Chronic	Poor bioavailability	Lacking human clinical trials for TBI; protective potential is demonstrated only in cellular and animal studies	Poor oral absorption; minimal BBB penetration; protective effects observed only in cellular and animal studies
Resveratrol	TBI, ICH	Preclinical; From cellular and animal studies	Cell culture and rodent models	Acute/Chronic	Nanoformulations improve delivery	Limited to cellular and animal studies, no large‐scale human clinical trials	Low drug bioavailability; brain‐targeted nanocarriers in early‐stage development; no human efficacy data
TREM2 modulation	TBI	Preclinical; From cellular and animal studies	Gene‐edited mouse injury models and in vitro microglia experiments	Subacute/Chronic	Genetic/pharmacological	Limited to basic mechanistic research without clinical translation	Unelucidated full TREM2‐lipid regulatory network; unknown human druggability; substantial barriers to clinical translation
LXR agonists	ICH, Stroke	Preclinical; From cellular and animal studies	Rodent models of cerebrovascular injury	Subacute/Chronic	Systemic	No human clinical trials targeting central disorders, research limited to animal studies	Broad interference with systemic lipid metabolism; risk of peripheral metabolic adverse reactions; undefined central therapeutic window
CYP46A1 activators	AD, Stroke	Preclinical; From cellular and animal studies	Transgenic AD mice and focal stroke animal models	Chronic	Crosses BBB	Merely a target for basic research, lacking human clinical validation	Lipid‐lowering and anti‐inflammatory effects verified merely in animals; uncertain human brain responses; no relevant clinical data
Ceramide synthase inhibitors	PD	Preclinical; From cellular and animal studies	Cultured neurons and PD animal models	Chronic	Systemic	Restricted to cellular and animal studies, with no progression to clinical trials	Poor ceramide synthase subtype selectivity; risk of disrupted human sphingolipid homeostasis; no human safety data
Orlistat	TBI	Preclinical; From cellular and animal studies	Rodent brain injury models with microglia	Acute/Subacute	Systemic	Only under mechanistic investigation in animals; absent human trials for neurological disorders, with approved clinical indication limited to peripheral weight reduction	Preliminary central lipid modulation in experimental animals; clinical use restricted to peripheral weight loss; unvalidated central efficacy in humans

In AD and PD, lipid abnormalities promote pathological protein deposition and neuronal degeneration. In SCI and TBI, lipid disorders dominate secondary injury, induce lipid peroxidation, foam cell formation, and abnormal mitophagy, exacerbate neurological damage, and impede tissue repair. In stroke and ICH, imbalanced lipid metabolism triggers atherosclerosis, BBB disruption, and energy metabolic dysfunction.

While the neuroprotective potential of various lipid‐regulating agents—such as statins, citicoline, and ferroptosis inhibitors—is promising in preclinical settings, their clinical translation remains fraught with challenges. First, the dynamic changes in lipid metabolism in the CNS across different disease stages have not been fully elucidated. For instance, cholesterol drives Aβ pathology in the early stage of AD, while it may participate in myelin repair at the late stage. In SCI, timely clearance of myelin cholesterol is critical for axonal regeneration, yet excessive cholesterol efflux may disrupt cell membrane integrity. Second, existing lipid‐targeted drugs cannot be efficiently and specifically delivered into the CNS, resulting in poor therapeutic effects. This also accounts for the clinical failure of traditional antioxidants in TBI and stroke trials. Third, lipid metabolism forms a complex regulatory network with other metabolic pathways. Accordingly, antioxidant‐based interventions alone are incapable of interrupting the entire pathological cascade.

In CNS diseases, lipid metabolism dysregulation not only affects individual cell types but also drives complex intercellular interactions that exacerbate pathological processes. Lipid peroxidation in neurons leads to the release of oxidative by‐products and debris, which activate microglia and drive them toward a pro‐inflammatory state characterized by lipid droplet accumulation and impaired phagocytic capacity. Microglial secretion of inflammatory cytokines, in turn, exacerbates neuronal oxidative stress and lipid dysregulation, creating a pathological feedback loop. Meanwhile, lipid metabolic dysfunction in neurons disrupts axon‐glial communication, impairing oligodendrocyte‐mediated myelin repair. Concurrently, oligodendrocyte dysfunction, marked by abnormalities in lipid metabolism and the release of oxidized lipids during demyelination, weakens myelin integrity and amplifies neuronal vulnerability. Microglia, influenced by lipid dysregulation, further release reactive oxygen species and inflammatory mediators that impede oligodendrocyte differentiation, leading to a vicious cycle of myelin damage and neuroinflammation. Additionally, cerebrovascular endothelial cells undergoing lipid abnormalities can compromise the blood–brain barrier (BBB), allowing peripheral lipid metabolites and inflammatory molecules to infiltrate brain tissue. These infiltrates activate microglia and exacerbate neuroinflammation. Simultaneously, neuron‐derived lipid metabolites can disrupt endothelial function, contributing to BBB breakdown in a bidirectional manner. Collectively, these intercellular interactions underscore the central role of lipid metabolism as a shared pathogenic mechanism linking various CNS cell types. Understanding these complex metabolic networks and their feedback loops is essential for identifying novel therapeutic targets and devising effective multitargeted treatment approaches.

Identifying novel lipid species as biomarkers moves precision medicine beyond traditional lipid panels, allowing us to capture the subtle metabolic dysregulation that drives complex diseases. Although these emerging lipid signatures show clear diagnostic and prognostic value in discovery cohorts, translating them into clinical practice requires overcoming several methodological barriers. A primary challenge is moving from research‐grade mass spectrometry to robust, high‐throughput clinical assays. This demands strict standardization of pre‐analytical variables and the development of targeted panels, such as those using multiple reaction monitoring (MRM), to guarantee reproducibility across different laboratories. Beyond analytical standardization, the real utility of lipidomics emerges through integration. Combining lipid profiles with genomic, proteomic, and clinical data will be necessary to build multiomic risk models that outperform conventional markers. Finally, establishing true clinical utility and causality will require validation in large‐scale, longitudinal prospective studies. Successfully navigating these steps will allow these lipid mediators to refine disease stratification and reveal new targets for personalized therapy.

In general, lipid metabolism represents a key shared mechanism in the pathogenesis of CNS diseases. Future research that deeply dissects its regulatory network, explores specific therapeutic targets, investigates combination interventions based on lipid metabolism, and translates basic findings into clinical practice may overcome current limitations in the treatment of CNS diseases.

## Author Contributions

Jiayan Yuan: conceptualization, writing‐original draft, visualization. Shixue Huang: writing – original draft, visualization. Kun Jiao: writing – original draft, visualization. Shu Yang: writing – original draft, visualization. Xuhui Ge: visualization. Wei Liu: funding. Wei Wang: conceptualization, writing – review and editing, supervision. Haoming Shu: conceptualization, writing – review and editing, supervision. Xuhui Zhou: conceptualization, writing – review and editing, supervision.

## Funding

This work was supported by the National Natural Science Foundation of China (Grant No. 82572867).

## Conflicts of Interest

The authors declare no conflicts of interest.

## Supporting information


**Figure S1:** Pathological roles of lipid dysregulation in AD. Neuronal LDs and impaired lipid metabolism promote APP processing by γ‐secretases, leading to Aβ plaque formation and mitochondrial oxidative stress. Meanwhile, APOE4 lipoproteins cross the BBB and activate microglia via TREM2 signaling, triggering pro‐inflammatory cytokines release and exacerbating neuroinflammation in AD. This figure was created by www.Biorender.com.
**Figure S2:** Lipid metabolism dysregulation in dopaminergic neurons and its role in PD. Astrocyte‐derived lipids, including those carried by APOE, are taken up by dopaminergic neurons. GBA1 mutations impair lysosomal function, disrupting cholesterol and PUFA metabolism, promoting α‐synuclein aggregation, lipid oxidation, and mitochondrial damage. Dysregulated lipid metabolic enzymes (e.g., ACSL4 overactivation and PLA_2_ downregulation) further drive neuronal lipid peroxidation and ferroptosis, contributing to dopaminergic neuron death in PD. This figure was created by www.Biorender.com.
**Figure S3:** Myelin debris‐induced lipid dysregulation and pathological cascade in SCI. Following SCI, myelin debris accumulates and is phagocytosed by microglia and macrophages, leading to cholesterol overload and foam cell formation. Impaired ABCA1‐mediated lipid efflux promotes lipid peroxidation, triggering ferroptosis in neurons and oligodendrocytes, while further amplifying neuroinflammation to exacerbate tissue damage. This figure was created by www.Biorender.com.
**Figure S4:** Mechanistic links between lipid metabolism dysregulation and TBI secondary injury cascade. TBI triggers the activation of cytosolic phospholipase A2 (cPLA2), which specifically degrades glycerophospholipids in the brain (such as PC and PE) and generates lysophospholipids (LPLs). The abnormal accumulation of lysophosphatidylcholine (Lyso‐PC) in the brain directly leads to neuronal apoptosis, axonal rupture, and synaptic dysfunction. Oxidative stress induces phospholipid degradation via cPLA_2_ activation, leading to cardiolipin peroxidation and subsequent mitochondrial dysfunction. Meanwhile, FASN acetylation promotes lipid droplet accumulation in microglia. These lipid abnormalities exacerbate mitochondrial damage, trigger neuroinflammation, and ultimately drive a detrimental secondary injury cascade resulting in neuronal cell death. This figure was created by www.Biorender.com.
**Figure S5:** Lipid metabolism in pathological cascades of stroke and ICH. In stroke, LDL‐C accumulation induces foam cell formation via HIF‐1α signaling, leading to vascular stenosis and thrombus formation. In ICH, red blood cell degradation triggers iron overload (Fe^2+^/Fe^3+^), elevating ROS, causing lipid peroxidation. Once BBB is damaged, lipid droplet accumulates in FABP4+ microglia cells, inhibiting the degradation of S100A9 protein, which leads to M1‐type microglia, thereby exacerbating neuroinflammation. Both diseases converge on shared core pathways: lipid peroxidation drives neuroinflammation, vascular endothelial damage, and neuronal death. This figure was created by www.Biorender.com.

## Data Availability

Data sharing is not applicable to this article as no new data were created or analyzed in this study.
