# Supplementary material for: Lipid Metabolism in Central Nervous System Diseases: Pathological Mechanisms and Therapeutic Advances
Source: CNS Neurosci Ther. 2026 Aug 25;32(8):e71109. doi: 10.1002/cns.71109 (PMC13507650; doi:10.1002/cns.71109)
Supplement: Supplementary file 1 [file CNS-32-e71109-s001.doc]

**Supplementary Figures and Figure legends**

**Figure S1.** **Pathological roles of lipid dysregulation in AD.** Neuronal LDs and impaired lipid metabolism promote APP processing by γ-secretases, leading to Aβ plaque formation and mitochondrial oxidative stress. Meanwhile, APOE4 lipoproteins cross the BBB and activate microglia via TREM2 signaling, triggering pro-inflammatory cytokines release and exacerbating neuroinflammation in AD. This figure was created by www.Biorender.com.

**Figure S2. Lipid metabolism dysregulation in dopaminergic neurons and its role in PD.** Astrocyte-derived lipids, including those carried by APOE, are taken up by dopaminergic neurons. GBA1 mutations impair lysosomal function, disrupting cholesterol and PUFA metabolism, promoting α-synuclein aggregation, lipid oxidation, and mitochondrial damage. Dysregulated lipid metabolic enzymes (e.g., ACSL4 overactivation and PLA_2_ downregulation) further drive neuronal lipid peroxidation and ferroptosis, contributing to dopaminergic neuron death in PD. This figure was created by www.Biorender.com.

**Figure S3. Myelin debris-induced lipid dysregulation and pathological cascade in SCI.** Following SCI, myelin debris accumulates and is phagocytosed by microglia and macrophages, leading to cholesterol overload and foam cell formation. Impaired ABCA1-mediated lipid efflux promotes lipid peroxidation, triggering ferroptosis in neurons and oligodendrocytes, while further amplifying neuroinflammation to exacerbate tissue damage. This figure was created by www.Biorender.com.

**Figure S4. Mechanistic links between lipid metabolism dysregulation and TBI secondary injury cascade.** TBI triggers the activation of cytosolic phospholipase A2 (cPLA2), which specifically degrades glycerophospholipids in the brain (such as PC and PE) and generates lysophospholipids (LPLs). The abnormal accumulation of lysophosphatidylcholine (Lyso-PC) in the brain directly leads to neuronal apoptosis, axonal rupture, and synaptic dysfunction. Oxidative stress induces phospholipid degradation via cPLA_2_ activation, leading to cardiolipin peroxidation and subsequent mitochondrial dysfunction. Meanwhile, FASN acetylation promotes lipid droplet accumulation in microglia. These lipid abnormalities exacerbate mitochondrial damage, trigger neuroinflammation, and ultimately drive a detrimental secondary injury cascade resulting in neuronal cell death. This figure was created by www.Biorender.com.

**Figure S5. Lipid metabolism in pathological cascades of stroke and ICH.** In stroke, LDL-C accumulation induces foam cell formation via HIF-1α signaling, leading to vascular stenosis and thrombus formation. In ICH, red blood cell degradation triggers iron overload (Fe^2+^/Fe^3+^), elevating ROS, causing lipid peroxidation. Once BBB is damaged, lipid droplet accumulates in FABP4+ microglia cells, inhibiting the degradation of S100A9 protein, which leads to M1-type microglia, thereby exacerbating neuroinflammation. Both diseases converge on shared core pathways: lipid peroxidation drives neuroinflammation, vascular endothelial damage, and neuronal death. This figure was created by www.Biorender.com.
